# The five tenets of family-based treatment for adolescent eating disorders

**DOI:** 10.1186/s40337-022-00585-y

**Published:** 2022-05-03

**Authors:** Renee D. Rienecke, Daniel Le Grange

**Affiliations:** 1 Eating Recovery Center and Pathlight Mood and Anxiety Centers, 333 N. Michigan Avenue, Ste. 1900, Chicago, IL 60601 USA; 2 Department of Psychiatry and Behavioral Sciences, Northwestern University, Chicago, IL USA; 3 Department of Psychiatry and Behavioral Sciences, University of California, San Francisco, CA USA; 4 Department of Psychiatry and Behavioral Neuroscience, The University of Chicago, Chicago, IL (Emeritus) USA

**Keywords:** Family-based treatment, FBT, Adolescents, Eating disorders, Key tenets

## Abstract

Family-based treatment (FBT) is the leading treatment for adolescent eating disorders and is based on five tenets, or fundamental assumptions: (1) the therapist holds an agnostic view of the cause of the illness; (2) the therapist takes a non-authoritarian stance in treatment; (3) parents are empowered to bring about the recovery of their child; (4) the eating disorder is separated from the patient and externalized; and (5) FBT utilizes a pragmatic approach to treatment. Learning these tenets is crucial to the correct practice and implementation of manualized FBT. The purpose of the current paper is to provide an in-depth overview of these five tenets and to illustrate how they are used in clinical practice. This overview will aid clinicians who are learning FBT.

## Introduction

Family-based treatment (FBT) has emerged as the leading evidence-based treatment for adolescents with eating disorders (EDs) and is recommended as the first-line treatment for patients who are medically stable for outpatient care [c.f., 1, 2]. The earliest studies of family therapy for anorexia nervosa (AN) were conducted at the Maudsley Hospital in London [3–5], with this approach subsequently adjusted somewhat in the United States, given a more behavioral focus, and called FBT [c.f., 6, for a description of FBT and the process of manualization]. These initial developments of FBT, building on the seminal work done at the Maudsley Hospital, occurred at The University of Chicago and Stanford University, leading to the clinician manuals for adolescents with AN, now in its second edition [7], and for adolescents with bulimia nervosa (BN) [8]. The early description of the manualization of FBT [6] briefly alludes to the fundamental assumptions of this treatment approach, that is, in FBT:

1. the therapist holds an agnostic view of the cause of the illness;
2. the therapist takes a non-authoritarian stance in treatment;
3. parents are empowered to bring about the recovery of their child;
4. the eating disorder is separated from the patient and externalized; and
5. FBT utilizes a pragmatic approach to treatment, with the focus on the here and now.

The purpose of the current paper is first to provide a more detailed overview of these five fundamental assumptions or key tenets of FBT, and second, to illustrate how they are used in clinical practice. As mentioned, although these tenets are briefly alluded to elsewhere [6], the current paper will provide an in-depth description of these tenets to aid in the training of therapists in the implementation of FBT and to improve dissemination of this important treatment approach.

### Overview of FBT

FBT consists of three phases: in Phase 1, parents are given the responsibility for bringing about weight restoration in the case of AN [7] or eliminating binge eating and purging and establishing a regular pattern of eating in the case of BN [8]. Parents are asked to make all eating-related decisions for their child, and usually curtail physical activity, until the ED has loosened its grip and is no longer influencing the child’s behaviors and thoughts about issues related to food, eating, shape, and weight. Due to the egosyntonic nature of AN, adolescent agency related to food is not a focus in the early stages of treatment, whereas Phase 1 for BN may allow for more of a collaborative approach between parents and children, due to the more egodystonic nature of BN. In Phase 2 of treatment, responsibility over eating is gradually handed back to the adolescent to whatever extent is age-appropriate and typical for that particular family. Phase 3 involves a review of healthy adolescence and an assessment of where the adolescent is developmentally once the ED has receded.

FBT is meant to be practiced by trained mental health providers experienced in the treatment of adolescents with EDs [7] and skilled in working with families. Given the medical complications and prevalent psychiatric comorbidities of these patients, a multidisciplinary team is essential. Other providers, such as physicians (pediatricians or general practitioners) and child and adolescent psychiatrists, can provide FBT-informed care and support the messages that the FBT therapist is giving, but the FBT therapist is the “leader of this clinical group” [7], p. 28].

## Tenets of FBT

The tenets of FBT are fundamental to the conduct and implementation of the treatment approach, but not all are described in detail in the treatment manual [7]. The following review is intended to serve as a guide for therapists learning FBT.

### Agnostic view of the illness

The central idea of this tenet is that treatment in the FBT model does not focus on exploring causes of the illness, but rather aims to engage the family as a resource to bring about early behavioral change. Eating disorders are complex illnesses, with genetic, sociocultural, personality/temperamental, and metabolic factors involved in their development [9, 10]. While family factors, such as weight-related teasing or maternal dieting, may increase the chances of developing unhealthy weight control behaviors or restrictive eating [11, 12] in those individuals who are vulnerable to developing an ED, the field has decisively moved away from blaming families for causing EDs, as there is no research to demonstrate that this is true [13]. Although there is evidence that families of patients with an ED report worse family functioning than healthy controls [14], this could very well be because of the negative impact of the ED on the family [15]. Thus, parents should not be viewed as a causal factor in the development of the illness, so no blame is assigned to parents in FBT.

Parents often feel to blame despite the FBT therapist assuring them that this is not the case. Although anecdotal, it is often the case that parents note that they must have ‘done something’ to cause the disorder, or they feel that they should have caught it earlier, which is easy to do given the often-insidious onset of the ED. It is important for the FBT therapist to address parental guilt, even if the parents do not bring it up themselves. Guilt can cause parents to second-guess themselves and doubt their ability to take appropriate action to solve the crisis of the ED. In addition, it can cause parents to lose confidence in their own ability to weight restore their child. Parents’ sense of self-efficacy in this domain has been shown to be a mediator of weight gain in FBT [16], and greater increases in parental self-efficacy during treatment predict greater adolescent weight gain [17]. Thus, parental confidence in FBT is very important. Anxiety, on the other hand, can be mobilizing provided it is not overwhelming. EDs are serious illnesses and parents should be at an optimal level of anxiety. The FBT therapist works to contain families who may be overly anxious, while raising the level of anxiety for those who are not “anxious enough” and thus may not be properly motivated to take on the task of weight restoration. However, it is important that the FBT therapist assures parents that there is no reason to feel guilty and, in fact, it is not a useful emotion in the context of this treatment.

Relatedly, FBT therapists do not pathologize the family. It is not helpful to think, for example, that one parent is somewhat controlling and that is why the adolescent developed AN. The FBT therapist keeps in mind that as a field we do not always know conclusively why EDs develop, and perhaps less so for any given individual. Furthermore, even if we did know why a particular person developed an ED, this knowledge does not necessarily translate to symptomatic improvement. Rather than spending time attempting to uncover the cause of the illness, FBT focuses on rapid symptom reduction. It is important to note that FBT is effective, not because it resolves a hypothetical family etiology, but rather because it changes the way a family responds to and manages their child’s ED.

*Maintaining an agnostic view in clinical practice*. One way to keep the focus of FBT agnostic is to appropriately address parents’ inevitable questions about why the ED developed. An FBT therapist might respond:

It is natural to want to know what caused the ED. EDs are complex illnesses with

several factors that come into play: genetic, environmental, personality, etc.

Unfortunately, we may never know for sure what caused the ED in your child’s

case. The good news is that we do not need to know what caused it in order to fix it.

One thing we do know is that parents are not to blame for the development of the ED. Rather, you will be an important resource – the most important resource - in your child’s recovery.

At times, parents have difficulty not pursuing this line of questioning, particularly if they firmly believe that the underlying cause of the ED needs to be identified for their child to recover. The “cancer analogy” [7] can be useful here:

I understand your desire to know what caused this illness. It feels like if you know

what caused it, you can keep it from coming back. However, we are in a crisis

situation and cannot afford to spend time hypothesizing about why the disorder

might have developed. If your son developed cancer, you would have it treated quickly

and aggressively, without waiting to find out what caused it. We are in a similar situation here. Spending time speculating about the cause of the eating disorder takes the focus off the hard work of behavioral change, which is what will help your son recover. Once the crisis is out of the way, we might be able to attend to the question of possible causes of your child’s eating disorder.

For the therapist, focusing on the family’s strengths rather than their weaknesses can be helpful in reducing any temptation to pathologize the family. Anecdotally, FBT therapists are often pleasantly surprised by the strength and creativity that families bring to the therapeutic process. Although the treatment is problem-focused, encouraging and acknowledging family strengths, while simultaneously working on ways that they can interact more effectively with their child and with the ED, will aid the therapist in keeping an agnostic perspective and not inadvertently blaming the family. Perhaps particularly important for new FBT therapists, supervision can also be useful in addressing any difficulties the therapist might be having in maintaining an agnostic view. Maintaining agnosticism is particularly important as it has been shown to be associated with recovery at the end of treatment. Lock et al. (2020) [18] found that fidelity to maintaining agnosticism at session 1 predicted recovery with 58% accuracy.

It is important to note that although the FBT therapist does not blame or pathologize the family, the FBT therapist should intervene when unhelpful or illness-maintaining family dynamics are observed. There is a great deal of research showing that high levels of parental expressed emotion, characterized by criticism, hostility, and emotional overinvolvement, result in poor treatment outcome [19]. When parents exhibit high expressed emotion, it can be useful to explore the reasons behind this. Are parents having difficulty externalizing the illness (reviewed below), resulting in attributing unwanted illness behavior to their child? Are parents burned out, and need assistance to problem-solve around utilizing additional resources in the weight restoration process, such as extended family members? It may be useful for an FBT therapist to say the following:

It is understandable that you may get angry when you see your child hides food or seems unable to complete a meal. Watching your daughter waste away is frightening for

parents, and this can sometimes come out as anger. However, we know that therapy suffers when parents get angry at their children. How can we help you manage this

anger and help you express the feelings that are actually behind it: concern and love?

If it is well-established in Phase 1, the agnostic view of the illness will generally carry over into Phases 2 and 3. If families are successful with FBT, their sense of self-efficacy grows, their feelings of guilt diminish, and they see that the ED is improving without the need to discuss the possible reasons behind its development.

### Non-authoritarian therapeutic stance

In FBT, the therapist is seen as an expert consultant. The therapist is the expert on EDs and on treatment, and the parents are seen as the experts on their child and their family. The therapist is active in treatment, providing psychoeducation and guidance, but generally does not tell the parents what to do. Or rather, parents are told what to do: “feed your child” but are not told exactly how to do this. In other words, therapists “give parents the frame, but the parents paint the picture”. Many of the decisions about how to implement treatment are left up to the parents. In addition to empowering parents, which will be reviewed shortly, it sends an important message to the child and to the ED that the parents are the primary agents of change in this treatment.

Many FBT therapists, even experienced ones, struggle with this concept. Taking a non-authoritarian therapeutic stance does not mean that parents are encouraged to do whatever they would like to do in treatment, or that the therapist can never take a more directive approach. Some of this decision-making may be based on how severe the adolescent’s situation is. For example, if an adolescent is at 78% expected body weight (EBW), actively restricting, and in Phase 1 of treatment, and parents want to send their child away to camp for several weeks, the therapist may start the conversation reviewing the pros and cons of such an action. If parents persist in this intended direction, the therapist may more firmly state that this is not in the best interest of the adolescent. However, ultimately the decision belongs to the parents. If they decide to send their child to camp, the therapist can then work with them to minimize the chances of a poor outcome, such as arranging regular weigh-ins at camp and having a camp nurse or counselor eat meals with the adolescent. On the other hand, if their child is near the end of Phase 2 and generally weight restored, the therapist can leave decision-making much more in the hands of the parents, with appropriate guidance. A similar approach is taken when discussing physical activity. For an adolescent who needs to weight restore, physical activity during Phase 1 is usually contraindicated and may even be medically unsafe. However, if a patient is medically stable and needs to gain weight, and the parents express their desire to continue physical activity for their child, a discussion of the pros and cons is warranted. The FBT therapist might point out that as it is, even without any physical activity, a substantial number of calories is needed to gain weight, and that this number will need to be increased even further if the child is exercising. Many patients are not willing to eat more to be allowed to exercise. However, if they are, and parents feel strongly about continuing physical activity, it can be useful to problem-solve with the parents in detail around how to increase caloric intake and ask them to consider discontinuing exercise if the patient loses weight or fails to gain at the expected tempo.

It is important for the FBT therapist to remember that there is no one-size-fits-all approach to this treatment. Therapists do not know all the family’s likes, dislikes, routines, habits, preferences, or details of their cultural, ethnic, or religious backgrounds—all of which can impact eating behavior. The therapist must believe that with his or her guidance, families will find their own answers that may be more effective than anything the therapist could prescribe.

*Maintaining a non-authoritarian stance in clinical practice*. To establish an FBT therapist’s role as an expert consultant, is it important that the therapist be knowledgeable about EDs and about the way in which adolescents with EDs think. Parents are often in a state of crisis when starting treatment, and they can be put at ease by seeing that their therapist is an expert on EDs, is up to date on the most recent research, has received training in FBT, and understands the mindset of an adolescent with an ED. Communicating to families that you understand the fear that their son or daughter experiences when faced with a plate of food, for example, can not only normalize their experience but may also aid in the development of rapport with the patient. An FBT therapist might say:

My role in treatment is to guide you through this process with the knowledge

I have about EDs and about this treatment approach. I will rely on you as the

experts on your child and your family as we work together to restore your child’s

health. It is important to note that AN is what’s sometimes called an ego-syntonic

illness, in contrast to an ego-dystonic illness. Anxiety and depression, for example, are ego-dystonic illnesses. If you are anxious or depressed, you don’t want to feel that way.

However, having an ego-syntonic illness means that part of you wants that illness.

Your daughter is ambivalent about treatment, so we cannot leave decisions about

recovery in her hands. Therefore, you will be playing a large role in treatment.

Maintaining this collaborative stance is an ongoing process. FBT therapists should reassure the family that although the primary responsibility for recovery is in the hands of the parents, the therapist will support them through this challenging journey until it is over.

Viewing parents as the experts on their family and their children, and giving them the responsibility for weight restoration, enables the family to reorganize itself to enhance parental effectiveness. Prior to presenting for treatment and over time, the family of a child with an ED tends to organize around and accommodate the illness [15] and the illness becomes the center of family life. Structural family therapy, one of the therapeutic approaches that FBT draws on [7], seeks to strengthen the parental subsystem and displace the ED from the central position it has come to occupy [c.f., 20. Although the ED may resist being “demoted”, the healthy part of the child often feels a sense of safety knowing that his or her parents are fighting the ED and making food-related decisions on behalf of their child, particularly given how difficult it is to fight the ED alone. This issue often presents in therapy when explaining why adolescents are not given choices about what to eat in Phase 1:

It makes sense that you would want to give your daughter choices about what to eat.

She is 16 years old and up until now she has had input into what she wants to eat.

However, when someone has an eating disorder, asking them to make a food-related

decision puts them in an internal battle – the healthy part of her versus the eating

disorder. This is an agonizing place to be, particularly when the eating disorder is

very strong. It is much kinder to temporarily take that decision away from her and shoulder that burden yourself. Your thinking is not clouded by the eating disorder and you know what is best for her right now.

The non-authoritarian therapeutic stance continues throughout Phases 2 and 3. It is often easier for FBT therapists to maintain this stance later in treatment as parents become more confident in their role and need less direct guidance from the therapist.

### Parental empowerment

Parental empowerment involves building confidence in parents to take on their role as the primary agents of change in the recovery process. To take on the challenging task of ensuring that an adolescent with AN eats an appropriate amount of food, parents have to have confidence that they can accomplish this task. Confidence enables parents to stand firm in the face of resistance from the ED, and to avoid second-guessing themselves when in difficult situations. If parents do not have confidence in themselves, the ED will realize this quickly and use it to its advantage, often in the form of negotiation around meals and snacks, to “poke holes in their parents’ armor”. Thus, it is vital that therapists communicate to the parents that they have confidence in the parents’ ability to take on this role. The therapist should remember that parents are the most important resource in therapy, as they are the ones who are with their child day in and day out and will be doing most of the work. It is also helpful for the therapist to remember that parents have valuable skills and strengths that they bring to treatment, that most parents can help their children renourish, and to never underestimate the love and concern that parents have for their children, which FBT harnesses so well. Like agnosticism, fidelity to establishing and maintaining externalization at session 3 has been found to predict recovery at end of treatment with 67% accuracy [18].

*Strategies for empowering parents in clinical practice*. As with taking a non-authoritarian stance, empowering the parents involves not telling them exactly how to go about the process of weight restoration. Therapists can give guidance and advice but encourage the family to come up with their own plan. This is one reason that prescribed meal plans are not used in FBT. By the time parents get to treatment, they have tried to battle the ED, either on their own or with other treatment attempts. They are often feeling quite helpless and defeated. To restore parents’ confidence in themselves, the FBT therapist might say the following:

Until the ED came along, you had a healthy child. You know how to feed your

child, but the ED has made you second guess yourself. Now you may feel that

you don’t know what to do, but you do. You have two other healthy children at

home, so you have the knowledge to do this. We are going to tap into that knowledge

so that you can restore your child to health.

Parental confidence can not only help parents stand firm in the face of resistance from the ED, but it also enables parents to handle difficult situations without feeling the need to ask for advice or support from their treatment team every time. Allowing parents to struggle at times through this process and come up with their own answers seems to be invaluable in increasing their confidence [21]. Giving parents too much guidance, including meal plans, would be in contradiction to the message that FBT therapists are trying to communicate. That is, FBT clinicians should be mindful not to tell parents that they know what to do *and* simultaneously give the message that they need to rely on an expert to get it done.

Parents sometimes genuinely feel that they do not have the nutritional knowledge necessary to bring about weight restoration. It is important for the FBT therapist to determine whether this is true, or whether the ED is making the parents doubt themselves in this domain. The following question can be extremely helpful in elucidating this:

You often hear about actors needing to bulk up for a movie role. If I asked you to

gain 30 pounds, would you be able to do it?

Parents almost inevitably answer yes, indicating that they are doubting their ability to refeed their child but do not lack the necessary nutritional information to do so. Parents are often concerned about what their child is *willing* to eat, rather than what he or she *should* eat. Pointing this out to parents can be helpful; when they no longer have to worry about buying the right low-calorie dressing for their child and they can instead focus on adequate nutrition, this can feel freeing and empowering. However, if parents are truly struggling with finding appropriately caloric foods, the therapist can provide examples of approaches that other families have found helpful or direct them to websites with high-calorie recipes.

Parents remain empowered through the latter phases of FBT. In Phase 2, parents retain oversight of eating behaviors while their child is developing more independence. For example, children may start to serve themselves dinner, but parents are there to add more if the child chooses an insufficient amount. In Phase 3, parents stay empowered in part by the reorganization of the family and the strengthening of the parental subsystem. Parents are the leaders of their own family and are no longer supplanted by the ED.

### Externalization of the illness

Separating the illness and the adolescent is a crucially important part of FBT. Anecdotally, when parents are struggling with treatment, it is often because they are having difficulty with this concept, so it is vital to ensure that parents understand it and successfully incorporate it into their conceptualization of the ED. Watching their child waste away due to eating too little evokes many emotions on the part of parents, including frustration, fear, worry, and anger. These are understandable reactions, but frustration and anger, especially when directed at the child, can be unhelpful. When their child sits at the dinner table and does not eat, parents must remember that their child is not being difficult, immature, or stubborn, and is not doing it on purpose. They must remember that the child is in the grip of a powerful disorder that is influencing his or her thoughts, feelings, and behaviors. The parents’ task is to battle the ED, not their healthy child, who is still there but may be overshadowed by the ED. Truly understanding this concept seems to be very effective in reducing parental criticism and hostility, which have been shown to have a negative impact on treatment retention and outcome [22, 23].

*Strategies for externalizing the illness in clinical practice*. There are several ways to explain this concept to families. One that is often used in FBT is to compare the ED to cancer or another serious medical illness. Like cancer, individuals with EDs do not choose to develop their illness, and once they have it, they cannot just decide to get better. Much as one cannot will away a tumor, their child cannot “just eat”. Their child has a serious illness and needs help to recover. Similarly, a caregiver would not become angry at their child or blame them for developing cancer. Likewise, children should not be blamed for developing the ED. Parents may still feel frustrated and angry by the circumstances, but these feelings should be directed toward the ED and not their ill child. FBT therapists may explain this in the following way:

At mealtimes it may seem that you are no longer interacting with your healthy, loving

child, and, in fact, you are not. You are dealing with a powerful disorder that has over-

taken your child, and she needs your help to overcome it. It is important to remain

compassionate with your daughter and remember how frightened she is to eat and gain weight, while remaining firm with the ED and ensuring that she gets the healthy amount of food that she needs. Parents sometimes find it helpful to name the ED, often “Ed”, to remind themselves that they are not fighting their daughter, but rather this terrible illness.

FBT therapists may also say to families that at mealtimes it may seem that they are interacting with an alien, or a being that has “possessed” their son or daughter. This often resonates with parents, particularly if resistance to eating is very strong and involves behaviors that are uncharacteristic of their healthy child, such as swearing or throwing food. This may also resonate with patients, who have described the ED as having “complete power over me” [24], p. 5]. Another helpful example comes from a television show seen by the second author, in which giant spiders jumped from trees, feeding on small birds foraging on the forest floor. The small birds struggled and fought as hard as they could but were unable to remove the spider from their backs. The only way to rescue these birds from such predatory spiders was for someone else to come along and take them off the birds. This is an apt metaphor for an ED. The child may struggle and fight against the disorder, but EDs are too strong for them to cast off themselves. They need their parents to come along and remove the ‘monster’. This example of externalization also seems to resonate well with parents.

However, there are times when despite repeated metaphors and examples, parents struggle to believe that their child needs their help, instead insisting that the child must want to get better for recovery to occur, and that they must fight the illness on their own. This is quite problematic; not only does it keep parents from assuming their necessary role in treatment, but, as mentioned above, it can cause parents to be angry and critical toward their child, thus having a negative impact on treatment outcome. FBT therapists may need to put in extra effort to help such families, moving forward with FBT only when externalization has been achieved. There are no clear guidelines as to when to abandon this treatment approach if parents are not in agreement with externalization or the other tenets of FBT. Early weight gain (approximately 4–5 pounds/2–2.5 kg by session 4) has been shown to predict good outcome in FBT [25–28]. If early weight gain does not occur, the therapist may consider another treatment modality, but clinical judgment is needed to determine whether the family simply needs more guidance and time, or whether they are not “buying into” FBT tenets and switching to another treatment is indicated. Research is needed to determine whether early weight gain is associated with parental agreement of FBT tenets.

Although externalization is a critical component of FBT, it should be used carefully, as patients can experience it as dismissive [24], and as if all their behaviors are being blamed on the ED. Engaging the healthy part of the adolescent in treatment as much as possible can aid in the family not losing sight of their healthy child’s identity.

Externalization often continues throughout the rest of FBT, although the need to continue to emphasize it with families perhaps becomes less pressing as they naturally incorporate this way of thinking into their language and behavior. It continues to be important during Phases 2 and 3 in part because externalization serves to de-pathologize the child and allows for the child to return to regular adolescent development. That is, the child does not need continued age-inappropriate supervision in the eating domain because he or she was just ill and is now recovering.

### Pragmatic approach to treatment

The main idea of this tenet is for the therapist to stay focused on the task at hand, which is symptom reduction. FBT is present- and symptom-focused with a strong behavioral approach. EDs are dangerous illnesses with high mortality rates [29] and potentially long-term medical consequences [30], even for adolescents, who presumably have shorter durations of illness than adults. Early, effective intervention is crucial to minimize any long-term damage that may occur and to prevent the ED from becoming more entrenched in the ill child’s personality, identity, and way of thinking. Because time is of the essence, and the goals of treatment are to help the child recover as quickly as possible and reduce the chances of developing a chronic disorder, FBT maintains a laser like focus on symptom reduction, particularly in the early phases of treatment.

Parents often ask that the therapist address issues that are secondary to the ED, including increased irritability, depression, anxiety, difficulty concentrating, or social withdrawal. Focusing on these other issues, which are likely to resolve with the resolution of the ED, takes the focus away from behavioral change, which is what will bring about weight restoration. It can be helpful to review the Minnesota Starvation Study [31] with families to educate them about the physical and psychological sequelae of EDs and the urgent need for rapid weight restoration to facilitate full recovery. So that parents do not feel ignored when expressing their concerns, it can be useful for the therapist to make note of the areas that the parents are worried about and assure them that these issues can be revisited in Phase 3, that is, when the crisis is over, and the child is well again. Often, when the issues are brought up again in Phase 3, it turns out they have been resolved and no longer need to be discussed.

*Maintaining a pragmatic approach in clinical practice*. In FBT, the therapist weighs the adolescent at the beginning of every session. The weight is then plotted on a chart, which is shared with the family. Starting sessions in this way sends a strong message to the family that weight is the focus of treatment, and indeed, weight loss or weight gain over the previous week sets the tone for each session. Similarly, in Phase 1, the therapist reviews meals and snacks in detail at every session, again reminding families that this is the focus of treatment for the time being. Parents may ask about other distressing issues or may wonder why their child is not receiving individual therapy to address, for example, poor body image or distorted thinking. The FBT therapist might respond:

While the ED has such a strong grip on your daughter, it is unlikely that she will

benefit from individual treatment. Furthermore, the ED is potentially deadly, whereas

poor body image is not. Our goal is to maintain our focus on adequate eating and

weight restoration. Research has shown us that these other symptoms you are seeing,

such as increased irritability and difficulty concentrating on schoolwork, will improve

as your daughter becomes more physically healthy. We must restore her physical

health first, and her emotional, mental, and psychological well-being will follow.

Remaining focused on the ED can be more challenging in Phases 2 and 3 of treatment, when the patient is out of immediate danger and may be progressing well. However, although the crisis has passed, there is still much work left to do in the process of recovery. Keeping the focus on eating behaviors allows the family to gradually navigate the return to normalcy while maintaining the gains they have made in Phase 1. Although it may be tempting to drift and move the focus of treatment to something more “interesting”, such as exploring possible underlying causes of the ED, this is not dictated by the manual, and there is no evidence that this will bring about speedier recovery for patients in FBT. Possible maintaining factors, such as overvaluation of shape and weight, are not addressed in FBT in the same way they are addressed in, for example, cognitive-behavioral therapy. FBT views family accommodation of the illness as a maintaining factor, which is disrupted with psychoeducation and reorganization of the family structure in their effort to address the ED. If FBT therapists feel that they are running out of topics or concerns to discuss when meeting with the family in latter phases, sessions may be scheduled less frequently.

Particularly for new FBT therapists, following the manual is another way to maintain the pragmatic approach to treatment. Anecdotally, therapists who are new to FBT often bemoan the fact that FBT can feel somewhat repetitive, especially in Phase 1, because of the firm focus on food and weight gain. However, the persistent focus on behavioral change in FBT may be one reason that it is so effective.

The purpose of this review of the FBT tenets is to provide guidance to those learning FBT and to aid in dissemination of this manualized treatment approach. Therapists learning FBT, as well as those who have been practicing FBT for some time, will find that the tenets serve as a “road map” to guide them in their clinical practice.

## Conclusion

The result of these five tenets of FBT is a focused treatment that emphasizes behavioral change rather than insight. Moreover, there is no direct focus on cognitive change through such cognitive-behavioral therapy techniques as cognitive restructuring, although cognitive improvement does occur because of treatment [32]. Although there is evidence that FBT therapists may “drift” from the manual and incorporate techniques or interventions from other treatment modalities, such as cognitive-behavioral therapy or dialectical behavior therapy [33], this is not recommended by the manual. FBT also results in improvements in family functioning [34]. These changes might perhaps be due to the way in which the family is ‘reorganized’ to support their unwell offspring, a process aided by psychoeducation and giving the parents responsibility for weight restoration, among other interventions.

The FBT manuals [7, 8] are valuable tools to guide clinicians in learning and implementing FBT. However, they cannot cover all possible situations that may arise in clinical practice. When faced with a clinical situation in which FBT therapists are unsure of which course to take, adhering to these five tenets will guide them to make the decision that will be most beneficial to the patients and their families. These tenets are inherent to the practice of FBT, and without following them, an FBT therapist is not practicing fidelity to the model.

Guidance such as that provided in the current paper may also aid in the dissemination of this important treatment approach. There is a limited number of FBT-trained therapists, thus, creative solutions are required to expand the availability of FBT to those who need it, such as delivering FBT via telehealth [35] or online training in FBT [18]. There is evidence that FBT can be successfully disseminated outside of the original treatment development sites [36–38], but much work remains to make FBT available to all families in need of support for their child with an ED.

In addition to access, the issue of manual adherence is an important one. Adherence to treatment manuals has been shown to improve patient outcomes in cognitive-behavioral therapy for BN [39]. However, research has found that many therapists do not practice FBT with fidelity. In a study of 117 clinicians who reported using FBT, Kosmerly et al. [40] found that one-third used techniques not recommended in the manual, such as individual therapy, mindfulness techniques, and motivational work. Another study of 40 therapists providing treatment for youth with AN found that the majority did not practice FBT with adherence, citing barriers such as discomfort with the family meal in the second session and lack of administrative support [41]. Although practicing a treatment with fidelity inherently seems a worthwhile goal, further research is needed on the relationship between treatment fidelity and treatment outcome. One study found that adherence to FBT was not related to weight gain, although treatment fidelity decreased over the course of therapy [42]. No other studies have examined adherence to FBT and patient outcomes; future research should further explore this topic using measures that have been developed to assess fidelity to this treatment approach [43]. Further, although taking a non-authoritarian therapeutic stance and taking a pragmatic approach to the illness are steeped in a rich history of family therapy approaches, they have not yet been shown to act as mediators of FBT and warrant future research attention.

## Data Availability

Not applicable.

## Abbreviations

AN: Anorexia nervosa
BN: Bulimia nervosa
EBW: Expected body weight
EDs: Eating disorders
FBT: Family-based treatment
